# Spatiotemporal dynamics of hippocampal-cortical networks underlying the unique phenomenological properties of trauma-related intrusive memories

**DOI:** 10.1038/s41380-024-02486-9

**Published:** 2024-03-07

**Authors:** Kevin J. Clancy, Quentin Devignes, Boyu Ren, Yara Pollmann, Sienna R. Nielsen, Kristin Howell, Poornima Kumar, Emily L. Belleau, Isabelle M. Rosso

**Affiliations:** 1 Center for Depression, Anxiety, and Stress Research, McLean Hospital, Belmont, MA USA; 2 Department of Psychiatry, Harvard Medical School, Boston, MA USA; 3 Laboratory for Psychiatric Biostatistics, McLean Hospital, Belmont, MA USA

**Keywords:** Psychiatric disorders, Neuroscience, Psychology

## Abstract

Trauma-related intrusive memories (TR-IMs) possess unique phenomenological properties that contribute to adverse post-traumatic outcomes, positioning them as critical intervention targets. However, transdiagnostic treatments for TR-IMs are scarce, as their underlying mechanisms have been investigated separate from their unique phenomenological properties. Extant models of more general episodic memory highlight dynamic hippocampal-cortical interactions that vary along the anterior-posterior axis of the hippocampus (HPC) to support different cognitive-affective and sensory-perceptual features of memory. Extending this work into the unique properties of TR-IMs, we conducted a study of eighty-four trauma-exposed adults who completed daily ecological momentary assessments of TR-IM properties followed by resting-state functional magnetic resonance imaging (rs-fMRI). Spatiotemporal dynamics of anterior and posterior hippocampal (a/pHPC)-cortical networks were assessed using co-activation pattern analysis to investigate their associations with different properties of TR-IMs. Emotional intensity of TR-IMs was inversely associated with the frequency and persistence of an aHPC-default mode network co-activation pattern. Conversely, sensory features of TR-IMs were associated with more frequent co-activation of the HPC with sensory cortices and the ventral attention network, and the reliving of TR-IMs in the “here-and-now” was associated with more persistent co-activation of the pHPC and the visual cortex. Notably, no associations were found between HPC-cortical network dynamics and conventional symptom measures, including TR-IM frequency or retrospective recall, underscoring the utility of ecological assessments of memory properties in identifying their neural substrates. These findings provide novel insights into the neural correlates of the unique features of TR-IMs that are critical for the development of individualized, transdiagnostic treatments for this pervasive, difficult-to-treat symptom.

## Introduction

Intrusive memories of a traumatic experience are common among trauma-exposed individuals and are predictors of the onset, maintenance, and severity of transdiagnostic post-traumatic sequalae [1–3]. As such, trauma-related intrusive memories (TR-IMs) are positioned as critical intervention targets [4]. However, mechanism-based treatments for TR-IMs are scarce, due in part to a lack of biological models that account for their unique phenomenological properties.

Transdiagnostically, TR-IMs are involuntary, spontaneous, and intrude on conscious thought. They are characterized by vivid sensory fragments that can emerge with deficient contextual details, such as place and time [5]. These sensory-perceptual properties of TR-IMs emerge on a continuum and can elicit a sense of reliving in the “here-and-now” [6, 7]. This reliving may distinguish TR-IMs from other forms of episodic memory and contributes to their dissociative counterpart – flashbacks – in posttraumatic stress disorder (PTSD) [8, 9]. Additionally, IMs exhibit distinct cognitive-affective properties, such as significant emotional distress and “attentional hijacking” [10, 11]. Various conceptual models of TR-IMs have emerged from these phenomenological properties, especially in relation to PTSD. The “warning signal hypothesis” emphasizes the exaggerated emotional intensity of TR-IMs as a learned cue to acquired threat, such that emotional qualities of TR-IMs may serve to activate threat-responding behaviors in response to a (mis)perceived threat cue [7, 12]. Another model, the “dual-representation theory”, expands on the sensory-perceptual features of TR-IMs, proposing two parallel, yet interacting, memory systems: (1) a higher-order contextual representation (C-rep) system that stores contextual details of the event, and (2) a lower-order sensory representation (S-rep) system that stores sensory-perceptual features [6, 13]. This theory suggests TR-IMs lack the necessary C-reps to bind exaggerated S-reps in place and time, thereby contributing to their sensory vividness and re-experiencing qualities. Overall, these models emphasize the critical role phenomenological properties play in understanding the underlying mechanisms of TR-IMs.

These conceptual models have inspired neurobiological accounts of TR-IMs that, while scarce, are grounded in decades of neurocognitive models of episodic memory [14]. The dual representation theory of TR-IMs positions a hyperactive sensory cortex and salience network (SN), consisting of the amygdala, dorsal anterior cingulate cortex (dACC), and anterior insula, as hubs of the exaggerated S-rep system. This is supported by accruing evidence that implicates the sensory cortex in trauma memory and intrusions [15–17] and the storage of conditioned threat in anxiety [18, 19]. Conversely, dysfunction of the hippocampus (HPC) is believed to underpin the deficient C-rep system, given the well-established role of the HPC in the contextual binding of memory details [20, 21]. Together, these systems are highly implicated in PTSD, in which TR-IMs are a central symptom [1, 14]. Canonical neural circuity models of PTSD emphasize a dysfunctional amygdala-hippocampal-medial prefrontal cortex circuit, specifically with regards to threat processing and conditioned fear [22]. More recent work has expanded this circuit to include disruptions in large-scale neural networks that consist of these structures, such as elevated activity within the SN [23, 24] and sensory systems [25], decreased connectivity in the default mode network (DMN) and HPC [24, 26–28], and a disruption in their anti-correlation [29]. Therefore, it stands to reason that such patterns of neural network activity may be associated with the experience of TR-IMs.

More broadly, episodic memory is associated with dynamic interactions between the HPC and large-scale networks, including the SN and DMN, and sensory, posterior-medial, and anterior-temporal systems [30–33]. These distributed networks support different aspects of episodic memory [34] and their functional segregations are mirrored in their distinct patterns of connectivity and co-activation with anterior and posterior segments of the HPC [33, 35–38]. Specifically, the posterior HPC (pHPC) has been linked to posterior-medial and sensory systems in supporting the detailed sensory-perceptual, particularly visuospatial, properties of memory and mental imagery [39, 40]. Regions of this posterior-medial system are also integral to the DMN, such as the precuneus, posterior cingulate cortex, and retrosplenial cortex, and are believed to contribute to the role of the DMN in autobiographical memory through mental imagery and the linking of sensory cues to mnemonic representations through interactions with the HPC [41, 42]. In contrast, the anterior HPC (aHPC) is predominantly connected to prefrontal and limbic structures supporting cognitive-affective features, including emotion and schematic gist [43, 44]. These structures consist of hubs of the SN (amygdala, dACC, insula) and DMN (mPFC), aligning with their respective functions in emotional processing and self-reference [45, 46]. Taken together, the dynamic interactions of these distributed networks with anterior-posterior divisions of the HPC are uniquely positioned to support the different cognitive-affective and sensory-perceptual properties of TR-IMs.

To date, the unique properties of TR-IMs have been investigated largely independently of their neurobiological substrates. The majority of neuroimaging research on TR-IMs has focused on the frequency and intensity of IMs, consistent with clinical assessments of the symptom, and implicate a diverse set of cortical regions linked to sensory, cognitive, and affective processes [17, 47–49]. Extensive work has implicated hubs of the SN and prefrontal cortex in both the encoding and retrieval of negative autobiographical and intrusive memories [50–52], consistent with their roles in threat processing, attentional control, and multimodal sensory integration [45]. However, there is a marked absence of studies examining the intrinsic neural correlates of the unique properties of TR-IMs that may distinguish them from general IMs.

Additionally, the HPC is often examined as a static and unitary structure in such research. Evidence is accruing for differential impacts of a/pHPC structure and function in PTSD [53–55], reflecting the functional heterogeneity of hippocampal subregions that may contribute to the diverse presentations of intrusion symptoms. Moreover, static connectivity measures between the unitary HPC and cortical structures may fail to capture the known dynamic nature of interactions between HPC subregions and distributed cortical systems [41]. The intrinsic dynamics of these HPC-cortical interactions is of particular relevance to clinical presentations, as TR-IM’s emerge spontaneously and intermittently from a “resting state” in a manner that is independent of volitional recall and often in the absence of conscious cueing.

Therefore, the objective of this study was to identify dynamic spatiotemporal patterns of intrinsic HPC-cortical co-activation that are associated with the different phenomenological properties of TR-IMs. We combined daily ecological momentary assessments (EMAs) of TR-IM properties with functional imaging of resting-state a/pHPC-cortical networks in trauma-exposed adults to test the hypothesis that the dynamics of different aHPC- and pHPC-cortical co-activation patterns would be associated with different TR-IM properties. Specifically, we hypothesized that the cognitive-affective properties would be associated with covariance of the aHPC with cognitive networks that regulate attention, self-reference, and emotion, including the SN and DMN. Additionally, we hypothesized that the sensory-perceptual properties would be associated with greater co-activation of the pHPC with the sensory cortex, posterior-medial networks, and the SN, given their implicated roles in the S-rep system of TR-IMs and demonstrated associations with IMs.

## Methods and materials

### Participants

Ninety-nine (99) trauma-exposed adults were recruited and enrolled via advertisements in the local community as part of a larger study. Study procedures were approved by the Mass General Brigham Human Research Committee and all participants provided written informed consent at Visit 1. Participants were recruited based on exposure to a Criterion A traumatic event and the endorsement of at least two TR-IMs per week over the past month, as defined by the DSM-5 [56]. Additional inclusion and exclusion criteria are provided in the Supplementary Information (SI).

Participants completed 2 weeks of daily EMAs of TR-IMs, after which they returned for Visit 2 to complete a clinical interview, self-report questionnaires, and a 13-minute eyes-open resting-state functional magnetic resonance imaging (rs-fMRI) scan. Of the 99 participants who completed the protocol, 84 had usable rs-fMRI data (excluded: excessive motion = 10, structural abnormalities = 2, poor structural-functional alignment = 1, falling asleep = 2). Demographic and clinical details are summarized in Table 1.

**Table 1:** Demographic and clinical characteristics. Means ± standard deviations or N (%).

Demographics (N = 84)	
Age (years)	31.1 ± 9.7
Race/Ethnicity (%)	
Asian	3 (4%)
Non-Hispanic Black	5 (6%)
Hispanic/Latino	1 (1%)
Non-Hispanic White	56 (67%)
Bi-/multiracial	16 (19%)
Missing	3 (4%)
Gender (%)	
Woman	58 (69%)
Man	15 (18%)
Non-binary	11 (13%)
Sex assigned at birth (female/male)	69/15
PTSD Diagnosis (%)	63 (75%)
CAPS-5 Total	33.7 ± 11.4
LEC-5 Total	12.1 ± 7.0
Total number of TR-IMs	23.1 ± 25.6

*CAPS-5* Clinician Administered PTSD Scale for DSM-5, *LEC-5* Life Events Checklist for DSM-5 [100].

### Ecological momentary assessments

Participants completed EMAs of the phenomenological properties of TR-IMs, which consisted of 3 daily surveys delivered on a semi-random schedule via the MetricWire smartphone app. Surveys assessed for the presence of TR-IMs since the last survey, followed by 18 prompts about their properties. Although participants were asked to identify and reference an index trauma at baseline, the EMA prompts did not specifically reference the “index trauma”, instead referencing “[their] trauma”. TR-IM properties were measured via prompts from the Autobiographical Memory Questionnaire (AMQ) [57, 58] and were rated on a 0-4 Likert scale. Ratings were grouped into vividness, visual detail, reliving (here-and-now), emotional intensity, fragmentation, and intrusiveness [59].

### Interview and self-report measures

#### Clinician-administered PTSD scale for DSM-5 (CAPS-5)

The CAPS-5 [60], the gold-standard diagnostic interview for PTSD, was administered by doctoral-level clinicians during Visit 2. This interview consists of 30 items assessing the onset, duration, and impact of PTSD symptoms, yielding a determination of PTSD diagnosis and symptom severity. Based on the CAPS-5, 75% of participants (*n* = 63) met diagnostic criteria for PTSD. Participants who met PTSD criteria did not differ on TR-IM properties compared to those who did not (*p*’s > 0.307). These group differences are detailed in the SI.

#### Autobiographical memory questionnaire (AMQ)

The AMQ [57, 58] is a 32-item questionnaire of autobiographical memory qualities, including vividness, visual features, other sensory features, bodily sensations, emotions, perspective, nowness, fragmentation, and intrusiveness. Participants completed this survey during Visit 2, retrospectively rating the qualities of their trauma-related memories on a 0-4 Likert scale.

### MRI data acquisition and preprocessing

Imaging was conducted at the McLean Hospital Imaging Center on a 3 T Siemens Prisma scanner with a 64-channel head coil. Structural and functional images were acquired using the Human Connectome Project (HCP) Young Lifespan protocols [61], including a 13-min eyes-open rs-fMRI scan. MRI data were preprocessed using fMRIPrep version 20.2.7 [62]. Additional preprocessing of rs-fMRI data was conducted using the CONN toolbox [63], including the removal of white matter and cerebrospinal fluid signals, scrubbing of motion outliers (FD > 0.5 mm), and high pass (0.01 Hz) filtering. Further protocol and preprocessing details are presented in the SI.

### Co-activation pattern analysis

Seed-based co-activation pattern (CAP) analysis [64] was used to compute spatiotemporal dynamics of a/pHPC networks. Relative to other well-validated dynamic connectivity methods (e.g., sliding window analysis), CAP analyses rely on fewer statistical assumptions and are not confounded by the sampling variability of fMRI that affects these other methods [65–67]. Additionally, CAP analyses may be able to capture more transient co-activation patterns given its higher temporal resolution [67].

a/pHPC seeds were defined based on prior work to maximize anterior-posterior segregation and minimize overlap with adjacent structures [53]. Analyses were performed using functions from the TbCAP Toolbox [68]. A union seed-based approach was used to identify volumes that exceeded an activation threshold of Z > 1 for either the aHPC, pHPC, or both to ensure only volumes characterized by HPC activation were evaluated [64, 69]. Spatial patterns of co-active regions within selected volumes were then clustered into co-activation patterns (CAPs) using k-means clustering. Consensus clustering was performed to determine the optimal number of CAPs within the data [70, 71] and identified *k* = 4 as optimal. Additional details on HPC seeds and clustering analyses are provided in the SI.

We computed the following CAP metrics within each participant: (1) *count*, reflecting the total number of supra-threshold volumes characterized by each CAP, which can be analogous to overall activity, and (2) *persistence*, reflecting the probability to remain in a given CAP across consecutive volumes, which can be analogous to stability. While individual differences in the total number of supra-threshold volumes in CAPs analyses can bias results [68], there were no associations between the total number of supra-threshold volumes across all CAPs and TR-IM properties (*p*’s > 0.07). Additionally, results were virtually identical when using fractional count, which controls for this individual difference in total number of supra-threshold volumes (SI Results).

### Statistical analyses

The cross-sectional associations between CAP metrics and TR-IM properties were evaluated using partial correlations of TR-IM ratings averaged across the EMA period, controlling for age and sex. Correction for multiple comparisons was performed using false discovery rate (FDR) at two levels – across all tests performed (4 CAPs x 6 TR-IM properties = 24 tests; FDR_total_) and across CAPs within each TR-IM property (4 CAPs x 1 TR-IM property = 4; FDR_property_). Properties demonstrating a significant effect were then entered as dependent variables in separate linear regression models with all CAPs as predictors to demonstrate a specificity of the association between individual TR-IM properties and CAPs. TR-IM properties and CAP metrics were mean-centered and scaled.

To evaluate the robustness of the cross-sectional results, repeated measures of TR-IM properties over the EMA period were analyzed by linear mixed models (LMM) with fixed effects for CAP metrics and a subject-specific random intercept to examine whether the same associations were present longitudinally. LMMs were used to account for the intra-subject correlations among the repeated measures. Two types of LMMs were considered: (1) univariate LMMs where CAP metrics were included individually and (2) multivariate LMMs where multiple CAP metrics were simultaneously included in the model. The analysis was performed in R version 4.2.2 (http://r-project.org/) using the lme4 package [72]. TR-IM properties and CAP metrics were mean-centered and scaled. The same FDR correction was applied to the results of univariate LMMs to account for multiple comparisons.

Finally, correlation analyses were performed between CAP metrics and conventional clinical measures, including total number of TR-IMs (frequency), retrospective reports of TR-IM properties at Visit 2, and CAPS-5 symptom severity, to demonstrate the utility of ecological assessments of TR-IMs. Additional analyses examining the moderating effect of PTSD diagnosis were performed to ascertain the disorder-specific nature of these effects.

## Results

### CAP characteristics

**Fig. 1:**
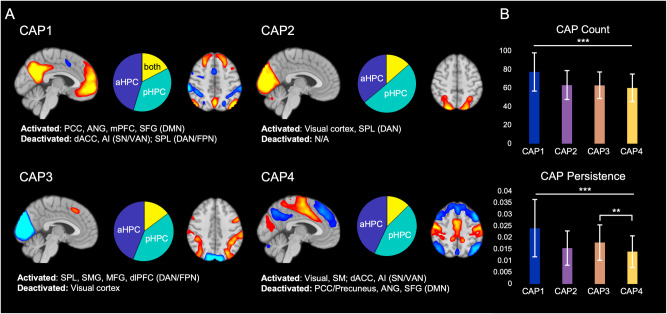
Representation of CAPs and their properties. (**A**) A summary of activated/deactivated regions and proportion of a/pHPC co-activation. (**B**) The average count and persistence of each CAP across participants. PCC posterior cingulate cortex, ANG angular gyrus, mPFC medial prefrontal cortex, SFG superior frontal gyrus, DMN default mode network, dACC dorsal anterior cingulate cortex, AI anterior insula, SN salience network, VAN ventral attention network, SPL superior parietal lobule, DAN dorsal attention network, FPN frontoparietal network, SMG supramarginal gyrus, MFG middle frontal gyrus, dlPFC dorsolateral prefrontal cortex, SM sensorimotor cortex. **p < 0.01, ***p < 0.001. Error bars reflect standard deviation.

The CAPs consisted of coactivation of the HPC with activation of the DMN and deactivation of the SN/VAN and DAN (CAP1), activation (CAP2) and deactivation (CAP3) of the visual cortex (VC), and a pattern of SN/VAN, VC, and sensorimotor activation with DMN deactivation (CAP4; Fig. 1A). CAP1 had more occurrences and persistence than all other CAPs (t’s > 3.82, *p*’s < 0.001; Fig. 1B).

Consistent with their divergent intrinsic connectivity networks, the aHPC and pHPC were differentially associated with the CAPs (Fig. 1A): CAP1 was predominantly associated with activation of the aHPC [mean volume counts (percentage volume counts) ± S.D. = 35.5 (45%) ± 11.1 vs. pHPC: 28.1 (37%) ± 8.1; t = 6.27, *p* < 0.001], while CAP2 was dominated by pHPC activation [32.2 (50%) ± 7.8 vs. aHPC: 22.7 (36%) ± 7.5; t = 11.3, *p* < 0.001]. CAP3 and CAP4 were associated with equivalent a/pHPC activation (*p*’s > 0.10).

### CAP count and TR-IM properties

**Fig. 2:**
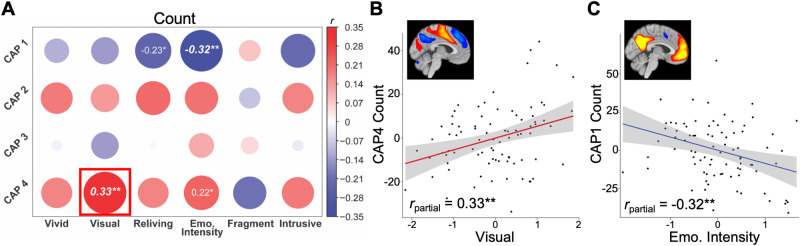
Associations between CAP count and TR-IM properties. **A** Partial correlations between count of all CAPs and TR-IM properties, controlling for age and sex, with the specific scatter plots of (**B**) CAP4 and visual features and (**C**) CAP1 and emotional intensity. Bold italics denote associations surviving correction for multiple comparisons. Boxes denote associations that were significant in multiple linear regression models, demonstrating specific association between that CAP and TR-IM property. * *p* < 0.05, ** *p* < 0.005.

Visual properties were associated with more occurrences of CAP4 (HPC–VC/SM/dACC/AI; *r*_partial_ = 0.33, *p* = 0.002, FDR_total_ *p* < 0.05; Fig. 2B), and emotional intensity was associated with fewer occurrences of CAP1 (aHPC-DMN; *r*_partial_ = -0.32, *p* = 0.003, FDR_total_ *p* < 0.05; Fig. 2C). Individual regressions for each significant TR-IM property testing the specificity of its associations with individual CAPs revealed a unique association between visual properties and CAP4 (b = 0.39, *t* = 2.69, *p* = 0.009) but did not demonstrate a specific association between emotional intensity and CAP1 (*p* = 0.668).

Results from univariate LMMs confirmed these associations – visual properties were associated with more CAP4 occurrences (b = 0.28, *t* = 3.11, *p* = 0.003, FDR_total_ *p* < 0.05) and emotional intensity was associated with fewer CAP1 occurrences (b = -0.23, *t* = -3.17, *p* = 0.002, FDR_total_ *p* < 0.05). Multivariate LMMs confirmed a specificity between visual properties and CAP4 (b = 0.37, *t* = 2.77, *p* = 0.007), but did not demonstrate a specificity between emotional intensity and CAP1 (*p* = 0.530; Table 2).

**Table 2:** Fixed effects of multivariate linear mixed effects models of significant TR-IM properties for all CAPs Count.

	Visual features	Emotional intensity
CAP1 Count	0.178 (0.171)	–0.090 (0.143)
CAP2 Count	0.212 (0.128)	0.074 (0.108)
CAP3 Count	0.096 (0.129)	0.099 (0.108)
CAP4 Count	0.369** (0.133)	0.176 (0.111)
Age	0.295*** (0.081)	0.117^†^ (0.068)
Sex	0.366 (0.228)	0.499* (0.192)
*Intercept*	–0.761^†^ (0.425)	–0.987** (0.358)

^*^p < 0.05, **p < 0.01, ***p < 0.001, †*p* < 0.1.

Estimates (*SE*).

### CAP Persistence and TR-IM Properties

**Fig. 3:**
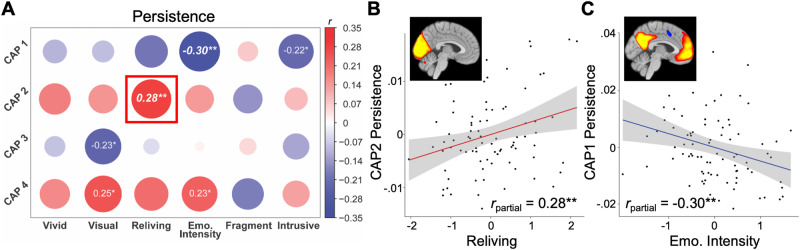
Associations between CAP Persistence and TR-IM properties. **A** Partial correlations between persistence of all CAPs and TR-IM properties, controlling for sex and age, with the specific scatter plots of (**B**) CAP2 persistence and reliving and (**C**) CAP1 persistence and emotional intensity. Italics denote associations surviving correction for multiple comparisons. Boxes denote associations that were significant in multiple linear regression models, demonstrating specific association between that CAP and TR-IM property. *p < 0.05, **p < 0.01.

Reliving was associated with more persistence of CAP2 (pHPC-VC; *r*_partial_ = 0.28, *p* = 0.009, FDR_reliving_ *p* < 0.05; Fig. 3B) and emotional intensity was associated with less persistence of CAP1 (aHPC-DMN; *r*_partial =_ –0.30, *p* = 0.007, FDR_emo_ < 0.05; Fig. 3C). Individual regressions confirmed a specificity between reliving and CAP2 (b = 0.25, *t* = 2.22, *p* = 0.029). The specificity between emotional intensity and CAP1 was non-significant (b = –13.29, *t* = –1.75, *p* = 0.084).

Results from univariate LMMs confirmed these associations – reliving was associated with CAP2 persistence (b = 0.21, *t* = 2.66, *p* = 0.009) and emotional intensity was associated with CAP1 persistence (b = -0.21, *t* = -2.86, *p* = 0.005). Multivariate LMMs confirmed a specificity between reliving and CAP2 (b = 0.20, *t* = 2.24, *p* = 0.028), but did not between emotional intensity and CAP1 (*p* = 0.108; Table 3).

**Table 3:** Fixed effects of multivariate linear mixed effects models of significant TR-IM properties for all CAPs Persistence.

	Reliving	Emotional intensity
CAP1 persistence	–0.017 (0.093)	–0.137 (0.084)
CAP2 persistence	0.197* (0.088)	0.053 (0.080)
CAP3 persistence	0.071 (0.098)	0.093 (0.088)
CAP4 persistence	0.187^†^ (0.103)	0.180^†^ (0.092)
Age	0.268*** (0.077)	0.128^†^ (0.070)
Sex	0.267 (0.211)	0.521** (0.191)
Intercept	–0.585 (0.392)	–1.025** (0.355)

^*^p < 0.05, **p < 0.01, ***p < 0.001, †*p* < 0.1.

Estimates (*SE*).

### Associations with conventional clinical assessments

No associations were found between CAPs and the total number of TR-IMs (absolute *r*’s < 0.12, *p*’s > 0.288). Similarly, no associations were found with PTSD symptom clusters or total PTSD symptom severity (absolute *r*’s < 0.15, *p*’s > 0.166). Additionally, there was no effect of PTSD diagnosis on the association between CAP metrics and TR-IMs (*p*’s > 0.140). Weak associations that did not survive FDR correction were seen with retrospective reports of TR-IMs – reliving was associated with fewer occurrences of CAP1 (*r* = -0.24, *p* = 0.031) and emotional intensity was associated with less persistence of CAP1 (*r* = -0.24, *p* = 0.034).

## Discussion

The primary aim of the present study was to utilize ecological assessments of the phenomenological properties of TR-IMs to shed light on their underlying neural correlates, specifically with regards to HPC-cortical interactions. Consistent with our hypotheses, divergent patterns of intrinsic HPC-cortical coactivation were associated with different TR-IM properties. Emotional intensity was associated with less frequent and persistent coactivation of the aHPC and DMN, while visual features were uniquely associated with more frequent coactivation of the HPC with sensory cortices and the VAN/SN. Additionally, reliving was associated with more persistent, but not frequent, co-activation of the pHPC and visual cortex. These findings align with prior work that demonstrates that different HPC-cortical systems support different multidimensional features of episodic memory. Moreover, our findings provide novel evidence for the involvement of these different systems in the unique properties of TR-IMs, which are a core symptom of PTSD.

To our knowledge, this is the first examination of CAPs in the context of PTSD and its symptomatology. CAP analyses have been increasingly used in the investigation of mechanistic processes underpinning various psychiatric disorders given their sensitivity to meaningful network dynamics [73–75]. Here, the use of CAP analyses allowed investigations into the dynamic patterns of co-activation between the HPC and cortical networks in a data-driven manner. The intrinsic functional architecture of the human brain is supported by evolving and dissolving “states” or patterns of coactivation that constitute canonical resting-state networks [65]. These canonical networks emerged within our data, including the DMN, D/VAN, SN, and visual network. The transient nature of these network configurations is believed to facilitate rapid and efficient information processing [76]. This function is of particular relevance for memory-related processes, given the widely distributed spatiotemporal networks involved in memory. It has been argued that static investigations of these networks averaged across time result in the loss of valuable information [64, 77, 78], thus emphasizing the importance of dynamic measures. Indeed, we found no associations between TR-IM properties and static functional connectivity of the HPC and identified CAP networks (p’s > 0.227; see SI). While some studies have shown that CAPs may not represent dynamic spatiotemporal properties of distinct network states [79], more recent work utilizing similar co-activation methodology has demonstrated meaningful temporal evolutions of network states that map onto temporally-varying behavioral processes [65]. Balancing these perspectives, we avoid the term “states” in reference to CAPs and discuss the frequency and persistence of these co-active patterns over time.

The most prominent and persistent co-activation pattern (CAP1) consisted of aHPC activation with the DMN and deactivation of attentional networks, including the VAN/SN, which is reflective of canonical “resting-state” activity. The integrity of the anticorrelation between the DMN and attention-related networks, like the VAN/SN, serves a critical role in supporting various cognitive-affective processes [80], and its disruption has been linked to numerous psychiatric disorders [29, 81, 82]. The DMN has gained increasing recognition in various cognitive processes, particularly memory. Hubs of the DMN are at the center of a “cortical memory retrieval network” [41] and are situated immediately downstream the HPC in a cascaded memory replay system [83]. The HPC and DMN demonstrate reciprocal interactions in the volitional retrieval of memory [32], supported by their robust intrinsic connections via the aHPC [36, 37]. The aHPC-DMN circuit in particular has been linked to the reconstructive recall of autobiographical memories, specifically the overall schematic “gist” [35]. Moreover, the core DMN system responds to the affective valence, but not vividness, of mentally reconstructed events [84] and both the aHPC and DMN contribute to the affective processing of past emotionally-laden experiences [85, 86]. Taken together, this aligns with our findings that link emotional intensity of TR-IMs to this aHPC-DMN CAP, suggesting this pattern of hippocampal-cortical interactions may be responsible for the affective features of autobiographical memory.

Although typically the aHPC and DMN interact in a facilitative manner to support these aspects of memory, the negative association we see here may reflect the pathological nature of TR-IMs, suggesting a memory process that is disrupted and contributes to exaggerated emotional intensity. This disruption is consistent with recent neuroimaging evidence implicating decreased connectivity between the aHPC and specific DMN subregions in PTSD symptom severity [54]. This emerges in the context of a rich literature on the neurocircuitry of PTSD that highlights decreased DMN activity and connectivity, often including the HPC [24, 26–28]. Together, a disruption in the frequency or stability of these aHPC-DMN interactions may reflect a breakdown in the intrinsic control of such affective memories, thus increasing susceptibility to spontaneous intrusions of the affective features of TR-IMs.

Additionally, the decreased frequency and stability of the deactivation of the SN at rest in CAP1 aligns, in part, with the “warning signal” hypothesis of TR-IMs, which emphasizes the role of conditioned threat in TR-IMs to facilitate attentional capture and orienting towards threat. Such rapid emotional processing and attentional capture is a critical function of the SN and a hallmark feature of TR-IMs. While the aHPC demonstrates robust structural connectivity with SN hubs [43], their intrinsic activity was characterized by co-deactivation, potentially reflecting the proposed regulatory role of the aHPC in such threat/emotion processing [38]. Failure to effectively deactivate the covariance of the SN with the aHPC may increase the vulnerability for affectively-laden threat memories to spontaneously emerge. This further aligns with prior work on the neural correlates of intrusive memories, which implicates SN activity in the emergence of intrusions [50–52] and increased connectivity between the aHPC and SN in the pathophysiology of PTSD [54].

Paralleling these affective properties, the sensory features of TR-IMs were associated with the frequency of co-activation of the sensory cortices and VAN/SN with the HPC (CAP4). Our probes of the sensory properties of TR-IMs focused on visual features given the predominant role of mental imagery in IMs [6]. While CAP4 was marked by co-activation of the visual cortex, there were similar activations across the somatosensory and motor cortices, reflecting multimodal sensory activity in relation to the sensory (visual) properties of TR-IMs. These findings are well-aligned with prior work demonstrating an active role of the broader sensory cortex in memory recall and formation [87–89]. CAP4 was also characterized by co-activation of the VAN/SN. As reviewed above, these networks are associated with bottom-up, sensory-driven attentional capture and are implicated in the processing of multimodal sensory stimuli [45]. Notably, both the sensory cortices and hubs of the VAN/SN have been theorized as neural substrates of the “sensory-representation system” in the dual-representation theory of IMs. Here, we provide critical evidence for the frequency of the co-activation of these networks in such sensory properties of TR-IMs and offer novel support for this facet of the dual-representation theory.

Surprisingly, the reliving properties of TR-IMs were associated with the persistence of co-activation of the visual cortex and the pHPC (CAP2). This CAP was hypothesized to support the sensory features of TR-IMs, given the role of pHPC-VC interactions in detailed mental imagery and recall of specific sensory details [35, 90]. Nonetheless, visuospatial details are known to contribute to the experiences of reliving the traumatic event in the here-and-now, characteristic of severe TR-IMs and their dissociative counterpart, “flashbacks” [91]. Moreover, extant models of flashbacks and the reliving of traumatic memories reliably implicate the visual system [52, 92], and reconstructive recall, or reexperiencing, of events is supported by the pHPC [30]. Indeed, pHPC interactions with visual areas have been found to support the elaboration, or mental reliving, of autobiographical memory through the recovery of sensory details [93]. It is important to note that the pHPC has also been positioned as a central structure regulating visuospatial contextual processing [35, 39], which, under the dual representation model, would minimize the experience of reliving in the “here-and-now”. While speculative, it is possible the exaggerated co-activation of these visuospatial contextual details (i.e., place) with exaggerated processing of visual details from the VC may actually exacerbate a sense of reliving through a “sensory replay” of visuospatial details. Moreover, the fact that this association emerged with persistence, and not frequency, of CAP2 could potentially reflect being “stuck” in a state of sensory processing of visuospatial contextual details, preventing the higher-order contextualization (i.e., time and autonoetic consciousness) through the integration of other cortical networks. Therefore, the persistence of co-activation between the pHPC and VC, even at rest, may bias the sensory-driven reconstructive recall of a traumatic event and contribute to the spontaneous reliving of TR-IMs. Notably, we did not aim to distinguish between ratings of TR-IMs and flashbacks in our EMA surveys. Some have argued the sense of nowness and reliving is a distinguishing factor between these two intrusion symptoms, while others suggest experiences of dissociation are more relevant [8, 9]. Therefore, additional studies examining the specific phenomenological details of reliving in TR-IMs, including dissociation, are needed to ascertain what elements of contextual processing are driving this sense of re-experiencing and if these processes are unique to flashbacks.

We did not find associations between HPC-cortical networks and TR-IM vividness or fragmentation. Interactions between the pHPC and posterior midline structures have been implicated in the vividness of memory recall and mental imagery [35, 39]. However, some data suggest the vividness of episodic memory may be mediated by cortical structures independent of the HPC, specifically the PCC/Precuneus, angular gyrus, and fusiform gyrus [42, 94, 95]. Conversely, the HPC has been more reliably implicated in the binding of episodic memory details into a coherent memory representation and is thus viewed as a hub for memory fragmentation, or lack thereof [21]. Therefore, it is possible unitary HPC dysfunction may underlie fragmentation, rather than its interactions with cortical networks. Alternatively, the role of HPC-cortical interactions in either vividness or fragmentation of memory may be task-dependent and not contingent on the intrinsic activity. Therefore, future studies utilizing task-based investigations of a/pHPC-cortical network dynamics are needed to ascertain their role in vividness and fragmentation.

Taken together, the sensory-perceptual properties of TR-IMs (i.e., visual features and reliving) were associated with more frequent and persistent activation of the HPC with sensory and bottom-up attention networks, which seems to contradict the dual-representation model that implicates decreased HPC activity in these networks [6]. Notably, some evidence suggests exaggerated contextual processing is linked to TR-IMs [96]. Balancing these viewpoints in speculation, the data here reflect a potential “hyper-co-activation” of these systems in TR-IMs, whereby the sensory processing of visuospatial contextual information heightens the visualization of trauma memories and contributes to the experience of reliving. Moreover, the co-activation of threat processing networks, such as the VAN/SN, in the implicated CAPs lends further credence to the “warning signal” hypothesis and the role of TR-IMs in threat-orienting behaviors through sensory-based, emotionally charged memories. Therefore, we propose the sensory-perceptual and cognitive-affective systems implicated in these models of TR-IMs are not disrupted independently, but rather interact in unique patterns to give rise to the diverse phenomenological experience of TR-IMs. Mechanistic studies probing these networks are needed to ascertain their distinct versus interactive patterns of activity in the emergence of TR-IMs.

With this in mind, the present study has a series of limitations. Notably, fMRI analyses were constrained to the resting state, and data were collected on the order of days to weeks after the completion of the EMA surveys. It is clear the ecological assessments of TR-IMs yielded valuable information, as no effects were seen with retrospective reports on the AMQ at Visit 2, which are susceptible to recall bias and loss of details – a well-known benefit of EMAs. However, we did not rigorously control which traumatic event participants referenced in their EMA surveys, therefore, it is possible the EMA surveys were completed in reference to other traumas in those with multiple Criterion A traumas, thus potentially increasing within-subject variability in reported TR-IM properties that may serve as a confounding factor. Additionally, our interest in the intrinsic activity of HPC-cortical networks was motivated by the spontaneous, “out of the blue” nature of TR-IMs, such that these intrinsic patterns of activity may provide insights into vulnerability to the spontaneous manifestation of TR-IMs. However, functional imaging of HPC-cortical network dynamics during memory retrieval may yield more rigorously controlled and nuanced insights into the neural substrates of these different memory processes. Specifically, the dynamic nature of CAP analyses would allow for identification of changes in neural “states” in response to spontaneous memories during a resting-state. Therefore, future studies examining these CAPs during either symptom provocation paradigms, prompted memory retrieval, or periodic probing for spontaneous memory emergence are warranted. Similarly, assessments of sensory-perceptual properties of TR-IMs beyond the visual system are needed, including somatosensory, auditory, and olfactory, as well as interoceptive sensations [16, 97]. Additionally, our sample was predominantly female, precluding any investigations into sex differences despite known effects of sex on PTSD symptoms. While we controlled for sex in our analyses, future studies matching groups by sex may yield more detailed insights into sex differences in the neurobiological substrates of TR-IMs.

Overall, our findings provide novel insights into the neural correlates of TR-IMs and elucidate unique neural network dynamics underpinning their phenomenological properties. The shared and unique co-activation patterns of the aHPC and pHPC lend further credence to their functional specialization with respect to large-scale neural networks and related aspects of memory. Their unique associations with different TR-IMs properties shed further light on previously observed heterogeneity in symptom-mechanism associations in trauma-related disorders. Moreover, our data demonstrate the clinical relevance of ecologically-valid assessments of the manifestation of intrusion symptoms, as no associations between TR-IMs and HPC CAPs were seen with retrospective recall measures. Together, these data position dynamic HPC-cortical networks as viable intervention targets for transdiagnostic TR-IMs. Indeed, recent developments of non-invasive brain stimulation and neurofeedback have successfully targeted the identified networks [98, 99]. The incorporation of these neuromodulatory techniques with detailed assessments of TR-IM properties may yield individualized, mechanism-based therapies for this pervasive yet difficult to treat symptom.

## Supplementary information

Supplemental Material

## Data Availability

Data are available through the NIMH National Data Archive (NDA; https://nda.nih.gov/edit_collection.html?id=3224) and are available upon reasonable request to the senior author, IMR. Shareable analysis code is available on the open science framework data repository (https://osf.io/exybv/).

## Code availability

Data are available through the NIMH National Data Archive (NDA; https://nda.nih.gov/edit_collection.html?id=3224) and are available upon reasonable request to the senior author, IMR. Shareable analysis code is available on the open science framework data repository (https://osf.io/exybv/).
