# Supplementary material for: Spatiotemporal dynamics of hippocampal-cortical networks underlying the unique phenomenological properties of trauma-related intrusive memories
Source: Mol Psychiatry. 2024 Mar 7;29(7):2161–9. doi: 10.1038/s41380-024-02486-9 (PMC11408261; doi:10.1038/s41380-024-02486-9)
Supplement: Supplementary file 1 — Supplemental Material [file 41380_2024_2486_MOESM1_ESM.docx]

**SUPPLEMENTARY INFORMATION**

*Spatiotemporal dynamics of hippocampal-cortical networks underlying the unique phenomenological properties of trauma-related intrusive memories – Clancy et al.*

**MATERIALS AND METHODS**

**Participants**

Inclusion criteria included ability to provide written informed consent, 18-65 years old, regular access to a smartphone to complete EMA surveys, and completion of 70% of daily EMA surveys. Participants were screened for TR-IM frequency and were considered eligible if they experienced 2 trauma-related intrusive memories (TR-IMs) in the past week. Exclusion criteria included left-handedness, medical conditions that would confound results, such as a seizure or other neurological disorder, history of moderate to severe traumatic brain injury, MR contraindications, including metal implants and claustrophobia, positive pregnancy test for female participants on the day of scanning. In addition, participants were excluded for current (past month) moderate-to-severe alcohol or substance use disorder, psychotic disorder, or manic or mixed mood episode. The present study utilized data from a larger study and focused only on participants with neuroimaging data. *A priori* power analyses for the larger study (NIH R01-MH120400) were set to detect a medium effect size (*r* = 0.3) with 80% power, requiring a total of n = 82 participants.

**Self-report measures**

*Life Events Checklist (LEC-5)*

The LEC-5 [1] is a 17-item assessment of potentially traumatic events used to determine which events a participant has experienced, witnessed, or learned about happening to a family member or close friend, reflecting a Criterion A trauma.

**MRI data acquisition and preprocessing**

MRI was conducted using the HCP Lifespan protocol [2]. T1-weighted 3D MPRAGE structural images were acquired using the HCP 0.8mm resolution sequence (TR/TEs: 2500/1.81/3.6/5.39/7.18; flip angle: 8 deg; FOV: 256 x 240; voxel size: 0.8mm isotropic), and eyes-open resting state T2-weighted echoplanar images were acquired using the HCP Lifespan sequence (TR/TE: 800/37 ms, in-plane resolution: 2mm; voxels: 2mm isotropic; multiband factor = 8; anterior-posterior phase encoding; one run of 976 frames, ~13 minutes in length).

T1-weighted (T1w) images were corrected for intensity non-uniformity [3]. Brain tissue segmentation of cerebrospinal fluid (CSF), white matter (WM) and gray matter (GM) was performed on the brain-extracted T1w [4]. Brain surfaces were reconstructed using recon-all [5], and the brain mask estimated previously was refined with a custom variation of the method to reconcile ANTs-derived and FreeSurfer-derived segmentations of the cortical GM of Mindboggle [6]. Volume-based spatial normalization to MNI standard space (MNI152NLin6Asym) was performed through nonlinear registration with antsRegistration (ANTs 2.3.3), using brain-extracted versions of both T1w reference and the T1w template.

EPI images were corrected for susceptibility distortions using the fMRIPrep fieldmap-less approach [7]. Based on the estimated susceptibility distortion, a corrected EPI (echo-planar imaging) reference was calculated for a more accurate co-registration with the anatomical reference. The reference was co-registered to the T1w reference with six degrees of freedom [8]. Head motion parameters with respect to the reference (transformation matrices, and six corresponding rotation and translation parameters) were estimated before any spatiotemporal filtering [9]. EPI images were slice-time corrected [10]. The time series were resampled onto their original, native space by applying a single, composite transform to correct for head motion and susceptibility distortions. The time series were resampled into standard space, generating a preprocessed run in MNI152NLin6Asym space. First, a reference volume and its skull-stripped version were generated using a custom methodology of fMRIPrep. Automatic removal of motion artifacts using independent component analysis (ICA-AROMA) [11] was performed on the preprocessed images on MNI space time-series after removal of non-steady state volumes and spatial smoothing with an isotropic, Gaussian kernel of 6mm FWHM (full-width half-maximum). Corresponding “non-aggressively” denoised runs were produced after such smoothing [11]. Additional preprocessing of resting-state fMRI data was conducted using the CONN toolbox [12], including the regression of physiological noise from white matter and cerebrospinal fluid using the CompCor method [13], scrubbing of motion outliers (FD > 0.5 mm) [14], and high pass (0.01 Hz) filtering.

Participants were excluded if their mean framewise displacement (FD) exceeded 0.5 mm or greater than 20% of volumes exceeded FD = 0.5 mm (n = 10) [14].

**a/pHPC ROIs**

a/pHPC ROIs were defined using the procedure outlined in Chen & Etkin (2013) [15]: HPC ROIs from the SPM12 Anatomy Toolbox were segmented along the anterior-posterior (Y) axis based on the following MNI coordinates: -10 to -21 (aHPC) and -32 to -43 (pHPC). These coordinates follow relevant gene-expression findings and anatomical landmarks, such as the uncal apex, while further controlling for overlap within HPC subregions and neighboring structures (i.e., amygdala).

**Co-activation Pattern Analysis**

Consensus clustering was run for *k* values of 2-11 based on prior work [16, 17] and theorized limits to the number of unique brain states at rest associated with significant co-activation of the HPC. Consensus clustering was run over 20 folds, each utilizing 80% of the data, for each k individually. For each *k*, a consensus quality was computed using 1 minus the proportion of ambiguously clustered pairs (PAC) [18], with higher values reflecting more consistent clustering across folds. As this quality index increases with the number of clusters used, we subtracted a fitted exponential function from the actual data to identify the *k* that most exceeded the expected trend [19, 20]. The combination of these techniques identified *k* = 4 as the optimal number of CAP networks (Supplementary Figure 1). K-means clustering was run 100 times to avoid local minima and ensure stability of the 4 CAPs.


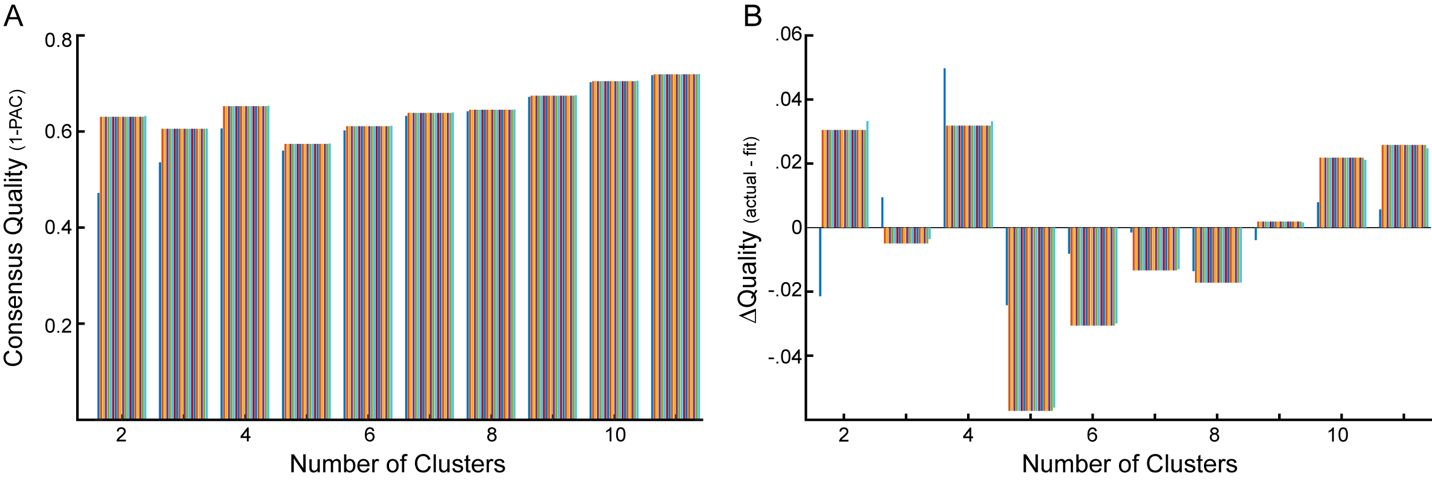


**Supplementary Figure 1.** CAP consensus clustering. A) Clustering quality as measured by 1 minus the proportion of ambiguously clustered pairs (PAC) across candidate cluster numbers. *k* = 4 stands as local peak, followed by expected exponential increase with increasing number of clusters. B) Subtracting a fitted exponential function from the quality index confirms *k* = 4 as optimal. The gradient displayed for each *k* value cluster denotes different criteria for defining “ambiguous clustering”, moving along a left-right gradient from less to more strict thresholding.

**Functional Connectivity**

ROI-to-ROI functional connectivity analyses were performed between a/pHPC ROIs and spatial masks generated from the identified CAPs to demonstrate the unique information achieved through dynamic connectivity analyses. This approach allowed us to hold the spatial information constant across these two analyses (dynamic vs. static), ensuring differences in results were driven by the temporal features. Binary spatial masks were generated for each individual CAP demonstrating significant associations with TR-IM properties, with separate masks generated for regions demonstrating either co-activation or co-deactivation, thresholded at Z = 1.5. Pearson correlation analyses were performed between a/pHPC or entire HPC ROI timeseries and the CAP timeseries, and then Fisher’s z-transformed prior to statistical analyses.

**RESULTS**

**PTSD vs. non-PTSD differences in TR-IMs**

There were no significant differences in TR-IM properties between participants with or without PTSD, based on the CAPS-5 Interview (p’s > 0.307; Supplementary Table 1).

**Supplementary Table 1.** TR-IM properties by PTSD groups. Means ± standard deviations.

|  | PTSD (n = 63) | No PTSD (n = 21) | Stats |
| --- | --- | --- | --- |
| Reliving | 1.65 ± 0.96 | 1.67 ± 0.89 | t = -0.09, p = 0.932 |
| Emo. Intensity | 2.32 ± 0.76 | 2.16 ± 0.91 | t = 0.72, p = 0.478 |
| Vision | 2.43 ± 0.96 | 2.25 ± 1.02 | t = 0.71, p = 0.483 |
| Vivid | 1.79 ± 0.93 | 1.61 ± 0.93 | t = 0.79, p = 0.435 |
| Intrusive | 2.67 ± 0.85 | 2.66 ± 0.90 | t = 0.05, p = 0.963 |
| Fragment | 2.09 ± 1.09 | 1.82 ± 1.05 | t = 1.04, p = 0.307 |
| Age | 31.92 ± 9.95 | 29.57 ± 8.70 | t = 1.03, p = 0.308 |
| Sex (female/male) | 51/12 | 18/3 | x^2^ = 0.03, p = 0.869 |

**Fractional count**

The average number of volumes exhibiting supra-threshold activation of the a/pHPC across participants was 264.2 volumes (SD = 11.8). Effects using fractional, instead of raw, CAP counts demonstrated virtually identical results. Visual properties were associated with more occurrences of CAP4 (*r*_partial_ = 0.29, *p* = 0.007), and emotional intensity was associated with fewer occurrences of CAP1 (*r*_partial_ = -0.34, *p* = 0.002).

**Static Functional Connectivity Effects**

There were no associations between TR-IM properties and HPC connectivity with the spatial CAPs (p’s > 0.227; Supplementary Figure 2).


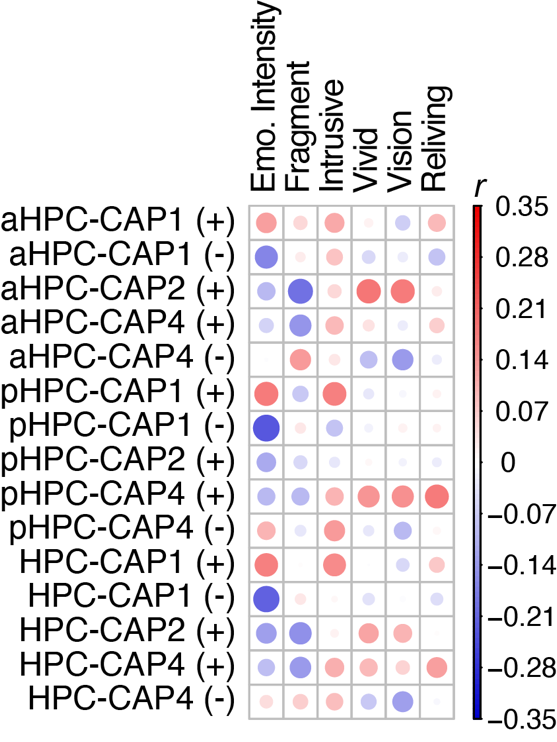


**Supplementary Figure 2.** Associations between TR-IM properties and static functional connectivity of the a/pHPC with spatial CAPs. Co-activation masks are indicated by (+) while co-deactivation masks are indicated by (-). Statistics reflect partial correlations, controlling for effects of age and sex. No effects reached statistical significance (p’s > 0.227).

**REFERENCES**

1. Gray MJ, Litz BT, Hsu JL, Lombardo TW. Psychometric properties of the life events checklist. Assessment. 2004;11:330–341.

2. Harms MP, Somerville LH, Ances BM, Andersson J, Barch DM, Bastiani M, et al. Extending the Human Connectome Project across ages: Imaging protocols for the Lifespan Development and Aging projects. Neuroimage. 2018;183:972–984.

3. Tustison NJ, Avants BB, Cook PA, Zheng Y, Egan A, Yushkevich PA, et al. N4ITK: improved N3 bias correction. IEEE Trans Med Imaging. 2010;29:1310–1320.

4. Zhang Y, Brady M, Smith S. Segmentation of brain MR images through a hidden Markov random field model and the expectation-maximization algorithm. IEEE Trans Med Imaging. 2001;20:45–57.

5. Dale AM, Fischl B, Sereno MI. Cortical surface-based analysis. I. Segmentation and surface reconstruction. Neuroimage. 1999;9:179–194.

6. Klein A, Ghosh SS, Bao FS, Giard J, Häme Y, Stavsky E, et al. Mindboggling morphometry of human brains. PLoS Comput Biol. 2017;13:e1005350.

7. Wang S, Peterson DJ, Gatenby JC, Li W, Grabowski TJ, Madhyastha TM. Evaluation of Field Map and Nonlinear Registration Methods for Correction of Susceptibility Artifacts in Diffusion MRI. Front Neuroinform. 2017;11:17.

8. Greve DN, Fischl B. Accurate and robust brain image alignment using boundary-based registration. Neuroimage. 2009;48:63–72.

9. Jenkinson M, Bannister P, Brady M, Smith S. Improved optimization for the robust and accurate linear registration and motion correction of brain images. Neuroimage. 2002;17:825–841.

10. Cox RW, Hyde JS. Software tools for analysis and visualization of fMRI data. NMR Biomed. 1997;10:171–178.

11. Pruim RHR, Mennes M, van Rooij D, Llera A, Buitelaar JK, Beckmann CF. ICA-AROMA: A robust ICA-based strategy for removing motion artifacts from fMRI data. Neuroimage. 2015;112:267–277.

12. Whitfield-Gabrieli S, Nieto-Castanon A. Conn: a functional connectivity toolbox for correlated and anticorrelated brain networks. Brain Connect. 2012;2:125–141.

13. Behzadi Y, Restom K, Liau J, Liu TT. A component based noise correction method (CompCor) for BOLD and perfusion based fMRI. Neuroimage. 2007;37:90–101.

14. Power JD, Barnes KA, Snyder AZ, Schlaggar BL, Petersen SE. Spurious but systematic correlations in functional connectivity MRI networks arise from subject motion. Neuroimage. 2012;59:2142–2154.

15. Chen AC, Etkin A. Hippocampal network connectivity and activation differentiates post-traumatic stress disorder from generalized anxiety disorder. Neuropsychopharmacology. 2013;38:1889–1898.

16. Kaiser RH, Kang MS, Lew Y, Van Der Feen J, Aguirre B, Clegg R, et al. Abnormal frontoinsular-default network dynamics in adolescent depression and rumination: a preliminary resting-state co-activation pattern analysis. Neuropsychopharmacology. 2019;44:1604–1612.

17. Chen JE, Chang C, Greicius MD, Glover GH. Introducing co-activation pattern metrics to quantify spontaneous brain network dynamics. NeuroImage. 2015;111:476–488.

18. Șenbabaoğlu Y, Michailidis G, Li JZ. Critical limitations of consensus clustering in class discovery. Sci Rep. 2014;4:6207.

19. Belleau EL, Bolton TAW, Kaiser RH, Clegg R, Cárdenas E, Goer F, et al. Resting state brain dynamics: Associations with childhood sexual abuse and major depressive disorder. NeuroImage: Clinical. 2022;36:103164.

20. Bolton TAW, Tuleasca C, Wotruba D, Rey G, Dhanis H, Gauthier B, et al. TbCAPs: A toolbox for co-activation pattern analysis. NeuroImage. 2020;211:116621.
